# Dynamic remodeling of the gut microbiome and host responses after myocardial infarction revealed by longitudinal metaproteomics

**DOI:** 10.3389/fmicb.2026.1826248

**Published:** 2026-07-10

**Authors:** Lvwen Ning, Zixi Chen, Hanchao Gao, Zhixiong Tan, Xiaoqing Quan, Lu Kong, Xiehui Chen

**Affiliations:** 1 Department of Geriatrics, Shenzhen Longhua District Central Hospital, Shenzhen, China; 2 Shenzhen Longhua District Key Laboratory of Personalized Precision Treatment for Elderly Coronary Heart Disease, Shenzhen, China; 3 Department of Nephrology, Shenzhen Longhua District Central Hospital, Shenzhen, China

**Keywords:** gut microbiota, gut–heart axis, host–microbe interactions, longitudinal analysis, metaproteomics, myocardial infarction

## Abstract

The gut microbiota is increasingly recognized as a key regulator of cardiovascular health; however, its functional dynamics following myocardial infarction (MI) remain poorly defined. While traditional sequencing approaches focus on microbial composition, they cannot capture real-time functional activity. In this study, we established a rat model of MI via permanent ligation of the left anterior descending artery and collected fecal samples from MI and sham-operated cohorts at baseline and Days 2, 7, and 14 post-surgery. High-resolution metaproteomics was applied to quantify microbial and host proteins, integrating functional annotation, differential expression, and weighted gene co-expression network analysis (WGCNA). We observed an acute, generalized decline in microbial diversity at Day 2 across both groups, indicative of a physiological response to surgical stress. Crucially, MI-specific divergence emerged during the subacute phase (Day 7) and persisted into recovery (Day 14). At Day 7, functional perturbations peaked in the MI group, significantly involving carbohydrate metabolism, nucleotide biosynthesis, and oxidative stress pathways, accompanied by taxonomic shifts including the depletion of Akkermansia and enrichment of Odoribacter and Muribaculum. Concurrently, host-derived proteins displayed time-dependent alterations in lipid catabolism and redox regulation. WGCNA revealed co-regulated protein modules linked to MI status and the recovery phase, reflecting highly synchronized host–microbiome responses. This time-resolved metaproteomic study demonstrates that following initial surgical stress, MI triggers dynamic, stage-specific alterations in gut microbial function and coordinated host responses, providing mechanistic insights into the gut–heart axis and suggesting potential microbiota-targeted strategies to promote post-MI recovery.

## Introduction

Myocardial infarction (MI) initiates profound systemic responses that extend far beyond the myocardium, engaging complex immune, metabolic, and neurohumoral pathways (Frangogiannis, 2015). Emerging evidence highlights the pivotal role of the gastrointestinal tract within this systemic reaction (Fathima, 2021), solidifying the concept of the gut–heart axis: a bidirectional communication network where the intestinal microbiota and their derived metabolites actively modulate cardiovascular health and disease pathogenesis (Jin et al., 2019; Abdulrahim et al., 2025; Snelson et al., 2025). Disruption of gut microbial homeostasis, or dysbiosis, is closely linked to compromised intestinal barrier integrity, amplified systemic inflammation, and exacerbated cardiovascular outcomes (Wang et al., 2011; Zhu et al., 2016; Katsimichas et al., 2019; Daniel et al., 2021; Xie et al., 2021; Abdulrahim et al., 2025; Yu et al., 2025). Conversely, specific bacterial taxa and key microbial metabolites, notably short-chain fatty acids (SCFAs) (Mansuri et al., 2022; Fan et al., 2023), have been shown to exert cardioprotective effects and preserve vascular function (Chambers et al., 2018; McDonald et al., 2018).

Despite growing recognition of the gut–heart axis, the majority of current research has relied on 16S rRNA sequencing or metagenomics to profile microbial composition (Hemmati et al., 2024; Matchado et al., 2024). While these DNA-based approaches offer excellent taxonomic resolution and reveal genetic potential, they fundamentally fail to capture the real-time, functional activity of the microbiome (Terrón-Camero et al., 2022). This methodological limitation obscures the dynamic functional shifts that occur within the gut ecosystem during acute pathological events like MI (Jovel et al., 2022; Terrón-Camero et al., 2022), where temporal alterations in microbial activity and host–microbe crosstalk are likely critical determinants of disease progression and recovery.

Metaproteomics overcomes this barrier by directly profiling the proteins actively expressed by both the microbial community and the host, offering a unique opportunity to decipher the molecular dialog at the host–microbiota interface (Wilmes and Bond, 2004; Karaduta et al., 2021; Stamboulian et al., 2022; Sun et al., 2024; Wang et al., 2024). Distinct from metagenomics or metatranscriptomics, metaproteomics delivers a precise snapshot of functional execution and host response at specific time points, facilitating the discovery of active pathways and protein-level interactions driving pathogenesis or recovery (Armengaud, 2023; Valles-Colomer et al., 2023; Wang et al., 2024). Furthermore, recent technological advances in high-throughput mass spectrometry, particularly data-independent acquisition (DIA), have dramatically enhanced the sensitivity, quantitative reproducibility, and depth of metaproteomic analyses, rendering it highly effective for dissecting complex host–microbiome networks (Petriz and Franco, 2017; Fröhlich et al., 2024; Pavelescu et al., 2024; Abdulaal et al., 2025).

In the present study, we applied longitudinal fecal metaproteomics to a rat MI model to systematically map the temporal dynamics of gut microbial composition, functional protein expression, and host–microbe co-regulation across the acute (Day 2), subacute (Day 7), and recovery (Day 14) phases following infarction. By integrating complementary microbial and host proteomic datasets, we aimed to delineate the dynamic, stage-specific shifts along the gut–heart axis and pinpoint specific functional pathways essential for post-MI recovery. Ultimately, this work provides novel mechanistic insights into the systemic reverberations of myocardial injury and establishes a critical foundation for developing appropriately timed, microbiota-targeted therapeutic interventions in cardiovascular disease.

## Materials and methods

### Animal model and study design

Twelve male Sprague–Dawley rats were obtained from Zhuhai Baishitong Biotechnology Co., Ltd. (Zhuhai, China; Animal Certificate No. 44822700033919, License No. SCXK (Yue) 2020-0051). Upon arrival (weighing 200–220 g), the animals were acclimatized for at least 1 week in a controlled environment (22 ± 2 °C, 50 ± 10% humidity, 12-h light/dark cycle) with *ad libitum* access to standard chow and water until they reached 250–300 g prior to surgery. MI was experimentally induced in six rats via permanent ligation of the left anterior descending (LAD) coronary artery, whereas six sham-operated control rats underwent an identical thoracotomy procedure without arterial ligation. Successful acute induction of ischemia was immediately verified by the observation of characteristic ST-segment elevation on continuous electrocardiography (ECG). Fecal samples were longitudinally collected from the subjects at baseline (Day 0; *n* = 3 per group to establish pre-surgical baseline variance) and post-surgery on Days 2, 7, and 14 (*n* = 6 per group).

### Cardiac functional and histological assessment

To comprehensively evaluate the progression of cardiac injury, transthoracic echocardiography was performed on Days 2, 7, and 14 post-surgery. Left ventricular ejection fraction (LVEF) and fractional shortening (LVFS) were measured to quantify functional impairment. At the study’s endpoint (Day 14), the animals were euthanized, and heart tissues were harvested, fixed in 4% paraformaldehyde, and embedded in paraffin. Serial cross-sections (4–5 μm) were subjected to Hematoxylin and Eosin (H&E) staining to evaluate morphological changes and inflammatory infiltration, and Masson’s trichrome staining to assess collagen deposition. For quantitative digital pathology analysis, whole-slide images of the Masson-stained sections were analyzed using QuPath software (version 0.7.0). A random trees pixel classifier was trained to differentiate viable myocardium, fibrotic tissue, and background based on color and texture features. Infarct size was subsequently calculated as the percentage of the fibrotic area relative to the total left ventricular tissue area.

### Fecal sample preparation and protein extraction

Microbial cell pellets were isolated through sequential centrifugation and subsequently lysed using a sodium dodecyl sulfate (SDS) buffer coupled with mechanical bead-beating to ensure robust cellular disruption. The extracted proteins underwent acetone precipitation, followed by reduction with tris(2-carboxyethyl)phosphine (TCEP) and alkylation using iodoacetamide (IAA). The protein samples were then digested overnight with trypsin at an enzyme-to-substrate ratio of 1:50 (w/w). Prior to liquid chromatography–tandem mass spectrometry (LC–MS/MS) analysis, the resulting peptides were desalted and purified via C18 solid-phase extraction.

### LC–MS/MS analysis

To maximize proteome coverage, the purified peptides were analyzed using two complementary mass spectrometry platforms. Data-dependent acquisition (DDA) was performed on an Orbitrap Exploris 480 (Thermo Fisher Scientific), while data-independent acquisition with parallel accumulation-serial fragmentation (DIA-PASEF) was executed using a timsTOF Pro (Bruker Daltonics). Peptides were chromatographically separated on C18 analytical columns using optimized solvent gradients. During the DDA runs, full MS scans were captured at a resolution of 120,000, accompanied by MS/MS scans at a resolution of 15,000. The DIA-PASEF acquisitions utilized 56 variable-width isolation windows combined with ion mobility separation to optimize both analytical sensitivity and quantitative accuracy.

### Proteomic data processing

Raw data files from the DDA platform were processed using MaxQuant software, whereas the DIA-PASEF data were analyzed with DIA-NN (version 1.8.1). Protein identification was matched against the UniProt rat reference proteome and a comprehensive, curated gut microbiome protein dataset (retrieved from https://ftp.cngb.org/pub/SciRAID/Microbiome/rat/GeneCatalog/rat_geneset.pep.gz). To ensure rigorous data quality control, the false discovery rate (FDR) was strictly set to < 1% at both the peptide and protein identification levels. Label-free quantification methods were utilized, and any proteins exhibiting greater than 20% missing values across the biological samples were systematically excluded from downstream analysis. Proteins were considered differentially expressed if they demonstrated an absolute log₂ fold change exceeding 1.5 and a *p*-value of less than 0.05.

### Functional and statistical analyses

The clusterProfiler package was employed for functional enrichment analyses, querying against the KEGG, COG, and MetaCyc databases. To evaluate microbial compositional shifts, alpha diversity was calculated using the Shannon, Chao1, and Simpson indices, while beta diversity was assessed via Bray–Curtis dissimilarity and visualized using Principal Coordinates Analysis (PCoA) and Non-Metric Multidimensional Scaling (NMDS). Group comparisons for beta diversity spatial clustering were statistically evaluated using PERMANOVA. Weighted gene co-expression network analysis (WGCNA) was implemented to integrate the host and microbial proteomic profiles, facilitating the identification of protein modules significantly associated with MI pathogenesis and subsequent recovery. General statistical evaluations incorporated one-way ANOVA, the Kruskal-Wallis H test, the Wilcoxon rank-sum test, and Spearman rank correlation as appropriate, with statistical significance established at *p* < 0.05.

## Results

### Validation of the myocardial infarction model and experimental design

Permanent ligation of the LAD coronary artery successfully induced MI in the experimental rat model. The acute ischemic event was immediately confirmed by characteristic ST-segment elevation on continuous electrocardiography. To comprehensively evaluate the progression of functional and structural cardiac injury, we conducted longitudinal echocardiographic monitoring alongside endpoint histological assessments. Echocardiographic analysis performed on Days 2, 7, and 14 revealed a significant and sustained reduction in both Left Ventricular Ejection Fraction (LVEF) and Fractional Shortening (LVFS) in the MI group compared to sham-operated controls (Supplementary Figure S1). At the study’s endpoint (Day 14), histological assessment via H&E staining revealed marked tissue remodeling, myocyte necrosis, and dense inflammatory cell infiltration within the infarcted myocardium (Supplementary Figure S2). Furthermore, quantitative digital pathology analysis of Masson’s trichrome-stained whole-heart cross-sections utilizing QuPath software demonstrated extensive transmural collagen deposition, confirming a substantial infarct size within the MI cohort (Supplementary Figure S3). In contrast, sham-operated animals exhibited no cardiac abnormalities throughout the study period.

**Figure 1:**
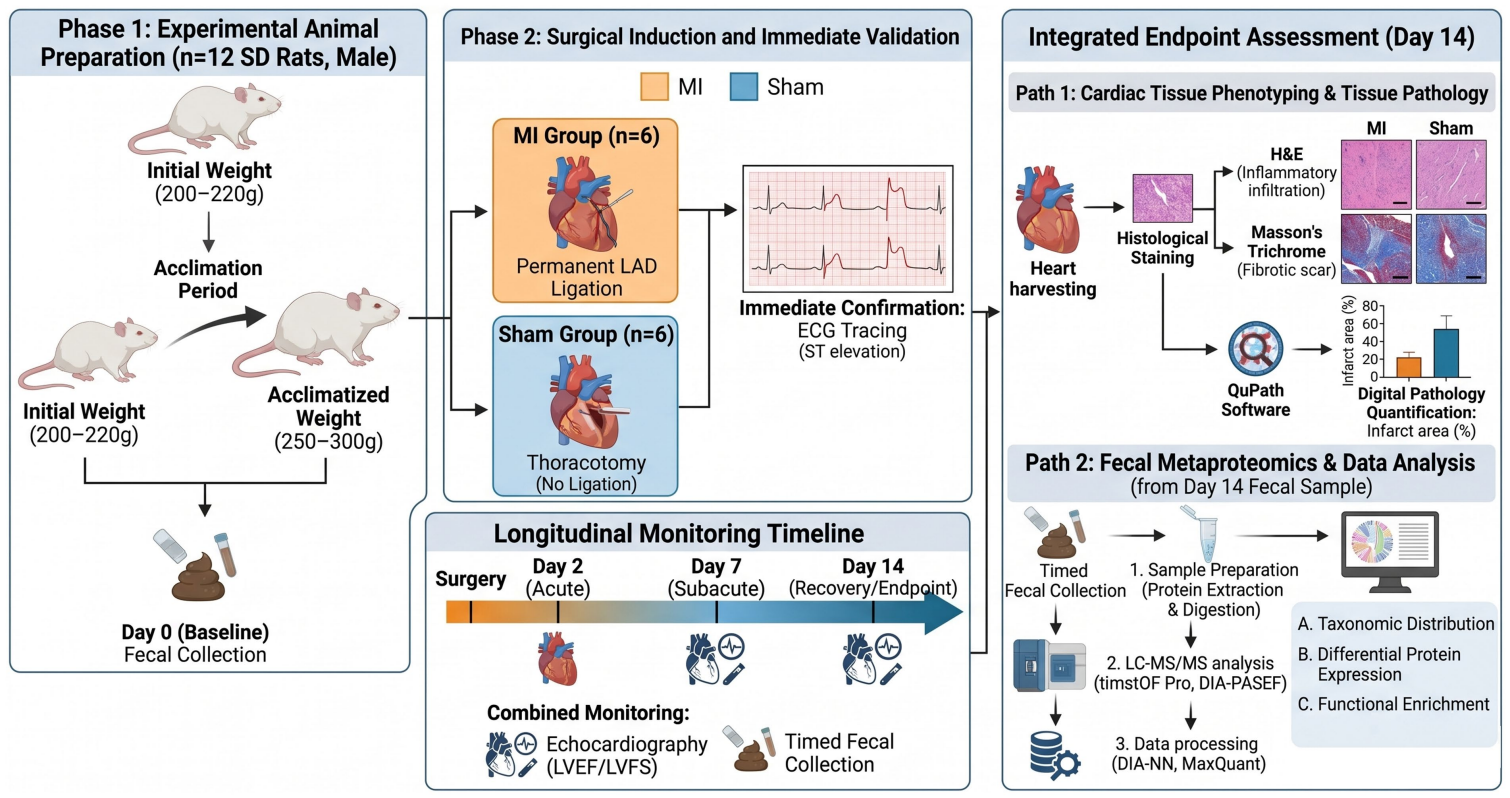
Schematic overview of the experimental design and metaproteomic workflow. The diagram illustrates the establishment of the rat myocardial infarction (MI) model via left anterior descending (LAD) coronary artery ligation, the longitudinal collection of fecal samples at baseline (Day 0) and post-surgery (Days 2, 7, and 14), and the subsequent high-resolution metaproteomic analysis pipeline for characterizing host–microbiome interactions.

Throughout the study, a total of 42 fecal samples were collected at baseline (Day 0) and on Days 2, 7, and 14 post-surgery from both the designated MI and sham cohorts (Day 0: *n* = 3 per group; Days 2, 7, and 14: *n* = 6 per group). A baseline sample size of *n* = 3 was utilized because these specific subjects adequately represented the pre-surgical baseline variation of the highly controlled, co-housed, and genetically homogenous experimental rat cohort. The comprehensive experimental workflow is illustrated in Figure 1.

### Taxonomic and functional landscape of the fecal metaproteome

**Figure 2:**
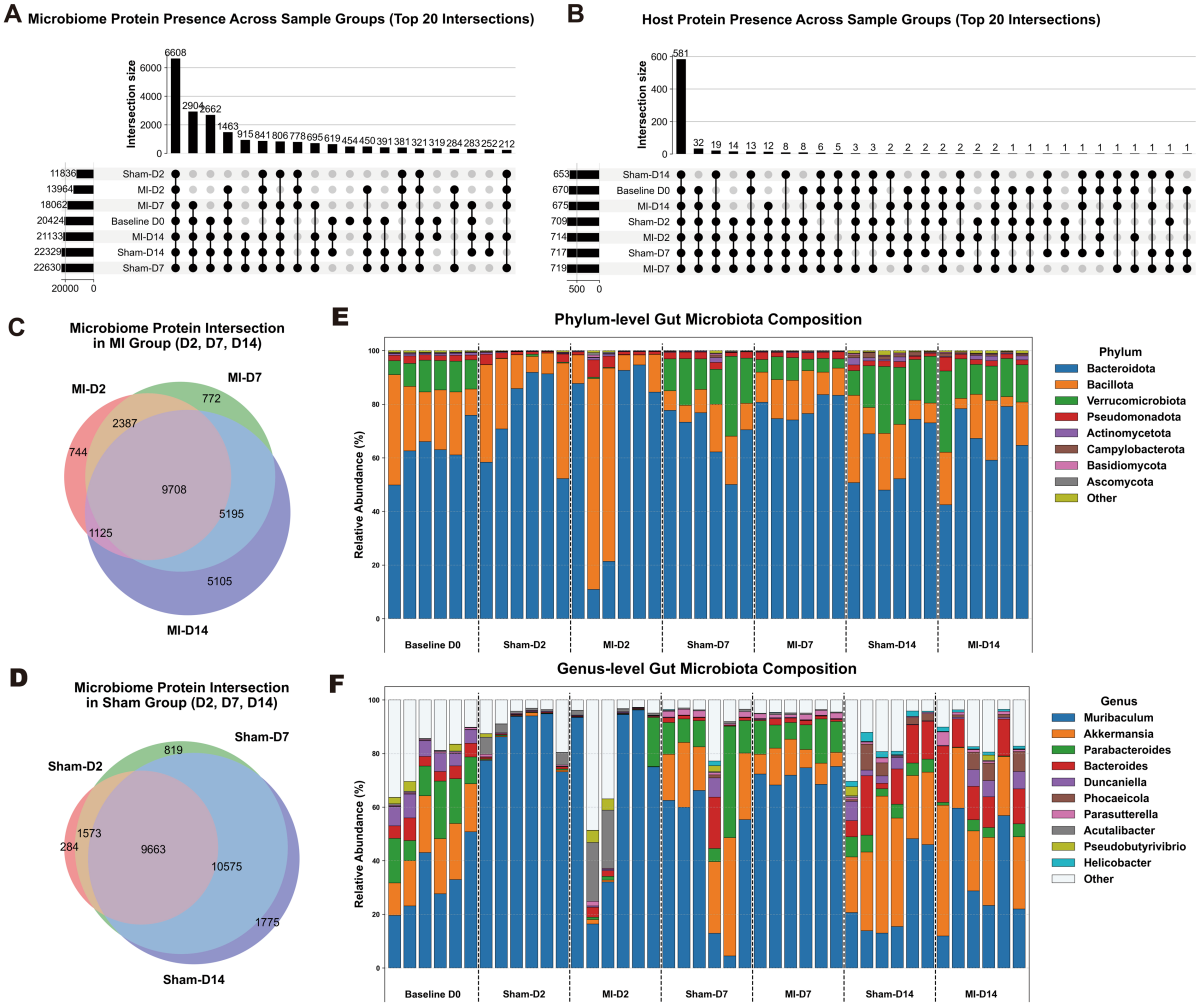
Taxonomic and functional landscape of the fecal metaproteome. **(A)** UpSet plot illustrating the presence and intersection of microbiome proteins across all experimental groups. **(B)** UpSet plot detailing the presence and intersection of host-derived proteins across sample groups. **(C,D)** Venn diagrams confirming the temporal reproducibility of the identified microbiome proteins across successive post-surgery time points (Days 2, 7, and 14) within the MI group **(C)** and the sham group **(D)**. **(E)** Phylum-level and **(F)** genus-level relative abundance of the gut microbiota depicting temporal compositional shifts.

High-resolution metaproteomic profiling yielded an extensive dataset comprising 33,663 nonredundant microbial proteins alongside 782 host-derived proteins. Over 6,000 microbial proteins were consistently detected across all experimental groups (Figure 2A), while nearly all host proteins were universally shared among the samples (Figure 2B). Furthermore, Venn diagrams confirmed that the majority of microbial proteins were reproducibly identified across successive time points in both the MI (Figure 2C) and sham groups (Figure 2D). Following rigorous quality filtering to exclude proteins with more than 20% missing values, 26,152 microbial and 729 host proteins were retained for robust quantitative analysis.

Taxonomic annotation of the recovered microbial peptides successfully assigned proteins to 37 phyla, 62 classes, and 366 genera, underscoring the profound microbial diversity present within the fecal proteome. At Baseline D0, the predominant phyla included Bacteroidota (63.1% on average), Bacillota (23.2%), Verrucomicrobiota (9.8%), and Pseudomonadota (2.2%) (Figure 2E). At the genus level, *Muribaculum* (32.9%), *Akkermansia* (18.3%), and *Parabacteroides* (13.9%) constituted the most abundant taxa, followed by *Bacteroides*, *Duncaniella*, *Phocaeicola*, *Parasutterella*, *Pseudobutyrivibrio* (Figure 2F). At the superkingdom classification, the vast majority of proteins (99.6%) were of bacterial origin, with marginal contributions from Eukaryota (0.29%), Archaea (0.08%), and viruses (0.006%). These proportional distributions highlight the bacterial dominance inherent in fecal proteomes while also suggesting that specific taxa, such as Verrucomicrobiota, might be systematically underrepresented in proteomic datasets when compared to their relative abundances typically determined by genomic sequencing.

To elucidate the functional capacity of the microbial communities, functional annotation of microbial proteins was performed utilizing both the KEGG Orthology (KO) and Clusters of Orthologous Groups (COG) databases. A total of 23,208 proteins were successfully mapped to 2,171 KEGG orthologs, and 22,297 proteins were assigned to 2,029 distinct COG categories. Level 3 KEGG analysis revealed that the highest proportion of proteins was devoted to metabolic functions—specifically, global and overview maps, carbohydrate metabolism, and energy metabolism. Within the domain of genetic information processing, the categories of translation, folding, sorting, and degradation were predominant, whereas signal transduction constituted the primary pathway within environmental information processing. Secondary functional contributions were attributed to cellular processes (notably signal transduction and cell motility), organismal systems (environmental adaptation and infectious disease), and human diseases (bacterial infectious disease and signal transduction) (Supplementary Figure S4A). The relative distribution of these primary KEGG functional categories across individual samples is depicted in Supplementary Figure S4B, demonstrating a highly consistent representation of core metabolic and information-processing activities throughout the microbiome.

COG classification revealed a similar pattern, with the most represented categories being J (translation, ribosomal structure, and biogenesis), G (carbohydrate metabolism and transport), and C (energy production and conversion) (Supplementary Figure S4C). Together, these annotations captured a broad spectrum of microbial functional capacities, including carbohydrate metabolism, amino acid biosynthesis, lipid metabolism, energy production, stress responses, and cell wall/membrane biogenesis, thereby providing a comprehensive overview of the gut microbial proteome.

Although host-derived proteins were numerically fewer than their microbial counterparts, they were significantly enriched in functional categories critical to immune regulation and epithelial barrier integrity, including defensins, calprotectin, and mucins. These host-specific protein signatures provide essential, complementary insights into the dynamic host–microbiome interactions and the evolving intestinal immune landscape over the course of post-MI recovery.

### Temporal dynamics of microbial diversity

To thoroughly characterize the temporal shifts in gut microbial diversity following MI and sham surgeries, we conducted a longitudinal analysis of fecal metaproteomic profiles at baseline (Day 0) and on Days 2, 7, and 14 post-surgery.

**Figure 3:**
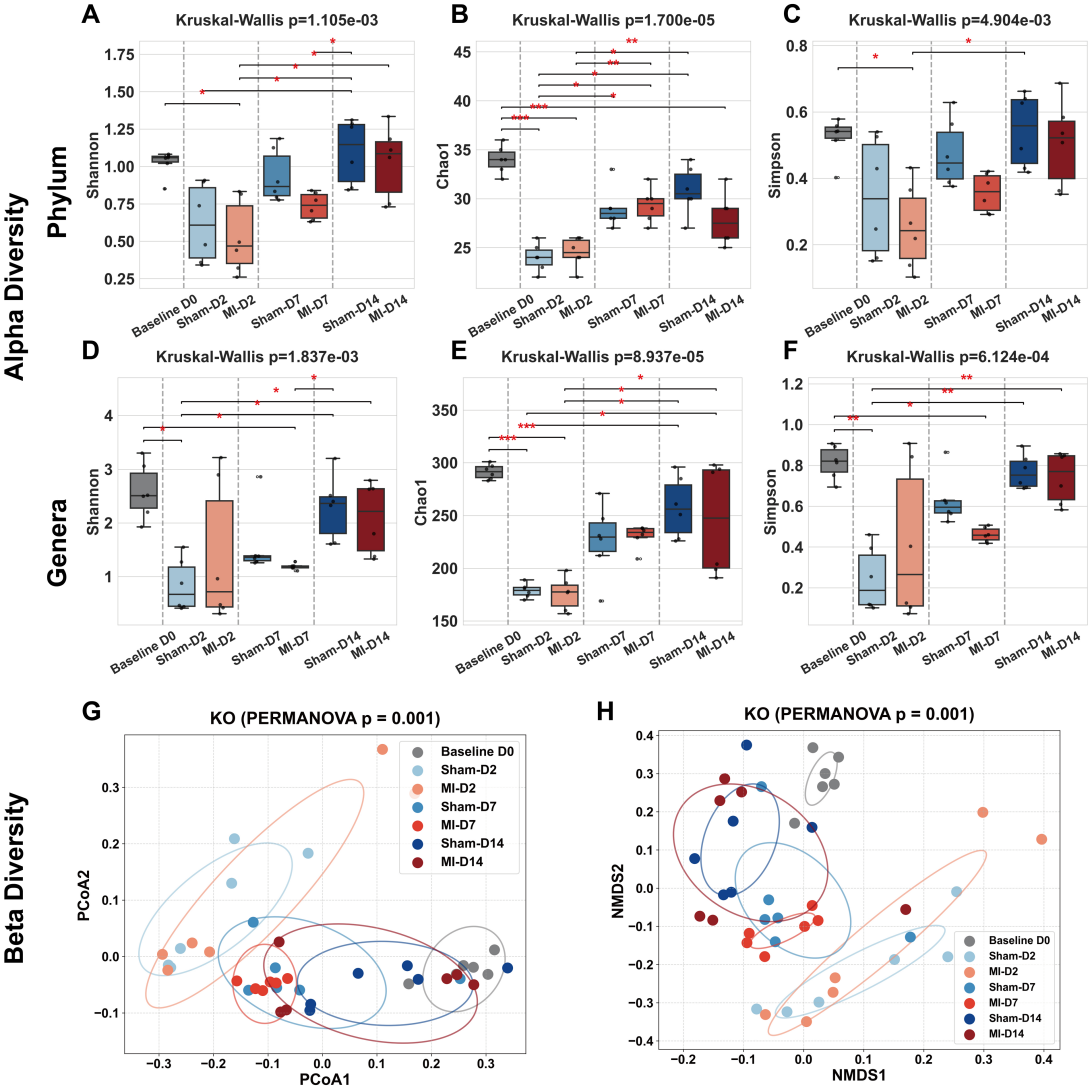
Longitudinal analysis of gut microbial diversity following myocardial infarction and sham surgery. **(A–C)** Alpha diversity metrics at the phylum level assessed using the Shannon index **(A)**, Chao1 index **(B)**, and Simpson index **(C)**. **(D–F)** Alpha diversity at the genus level assessed using the same indices: Shannon **(D)**, Chao1 **(E)**, and Simpson **(F)**. All indices demonstrate a significant reduction in microbial diversity at Day 2 (D2) in both the MI and sham groups, followed by partial recovery at Day 7 (D7) and near-complete restoration by Day 14 (D14). **(G)** Principal Coordinates Analysis (PCoA) and **(H)** Non-Metric Multidimensional Scaling (NMDS) based on Bray–Curtis dissimilarity reveal distinct temporal clustering of microbial communities, highlighting a clear shift on D2 and progressive convergence toward the Baseline D0 composition by D14. Statistical significance was assessed using appropriate tests with correction for multiple comparisons.

Alpha diversity—evaluated via the Shannon, Chao1, and Simpson indices—exhibited a precipitous and statistically significant decline at Day 2 in both the MI and sham-operated cohorts relative to baseline (Figures 3A–F). Given the lack of significant difference between the two groups at this initial time point, this acute loss of microbial richness and community evenness primarily reflects a generalized physiological response to surgical trauma and anesthesia. By Day 7, microbial diversity demonstrated a partial rebound. Notably, the sham cohort exhibited a tendency toward recovery during this subacute phase, whereas the MI group displayed a relatively delayed restructuring. By Day 14, both groups eventually approached baseline values, albeit with minor residual perturbations.

Furthermore, beta diversity analysis underscored a profound temporal restructuring of the microbial communities. Both Principal Coordinates Analysis (PCoA) and Non-Metric Multidimensional Scaling (NMDS), utilizing Bray–Curtis dissimilarity metrics, revealed a distinct and marked clustering of the Day 2 samples away from baseline profiles, visually reflecting the acute post-surgical community shifts (Figures 3G,H). Crucially, group comparisons utilizing PERMANOVA confirmed statistically significant differences in community composition across these temporal stages (*p* = 0.001). The Day 7 samples occupied an intermediate spatial position, highlighting the divergence in recovery trajectories between groups, whereas the Day 14 samples largely overlapped with the baseline clusters, indicating a near-complete restoration of the broad community composition.

Taken together, these findings demonstrate that gut microbial diversity undergoes an acute, non-specific collapse immediately following the physiological stress of surgery, which is then followed by a progressive recovery trajectory. The divergence in recovery rates between the MI and sham groups starting at Day 7 highlights the emergence of MI-specific microbial remodeling following the initial shared stress response.

### Taxonomic alterations across groups and time points

To evaluate the taxonomic alterations associated with MI and sham surgeries, microbial profiles were compared across all experimental groups and time points utilizing Kruskal–Wallis H tests. Analysis of the 37 annotated phyla revealed that 14 exhibited significant temporal dynamics (FDR < 0.05; Table 1).

**Table 1:** Significant changed phylum across groups by metaproteins.

Kingdom	Phylum	Statistic	*P* value	FDR
Bacteria	Verrucomicrobiota	30.07	3.80E-05	1.41E-03
Bacteria	Campylobacterota	24.58	4.08E-04	7.55E-03
Bacteria	Deferribacterota	23.62	6.13E-04	7.56E-03
Bacteria	Bdellovibrionota	21.99	1.21E-03	1.12E-02
Eukaryota	Zoopagomycota	21.47	1.51E-03	1.12E-02
Archaea	Candidatus_Helarchaeota	20.56	2.20E-03	1.22E-02
Bacteria	Thermodesulfobacteriota	20.44	2.31E-03	1.22E-02
Archaea	Thermoproteota	18.03	6.15E-03	2.53E-02
Eukaryota	Mucoromycota	18.27	5.59E-03	2.53E-02
Bacteria	Lentisphaerota	17.63	7.22E-03	2.67E-02
Bacteria	Spirochaetota	16.87	9.79E-03	3.29E-02
Archaea	Candidatus_Woesearchaeota	15.57	1.63E-02	4.63E-02
Bacteria	Aquificota	15.58	1.62E-02	4.63E-02
Bacteria	Planctomycetota	15.19	1.89E-02	4.99E-02

**Figure 4:**
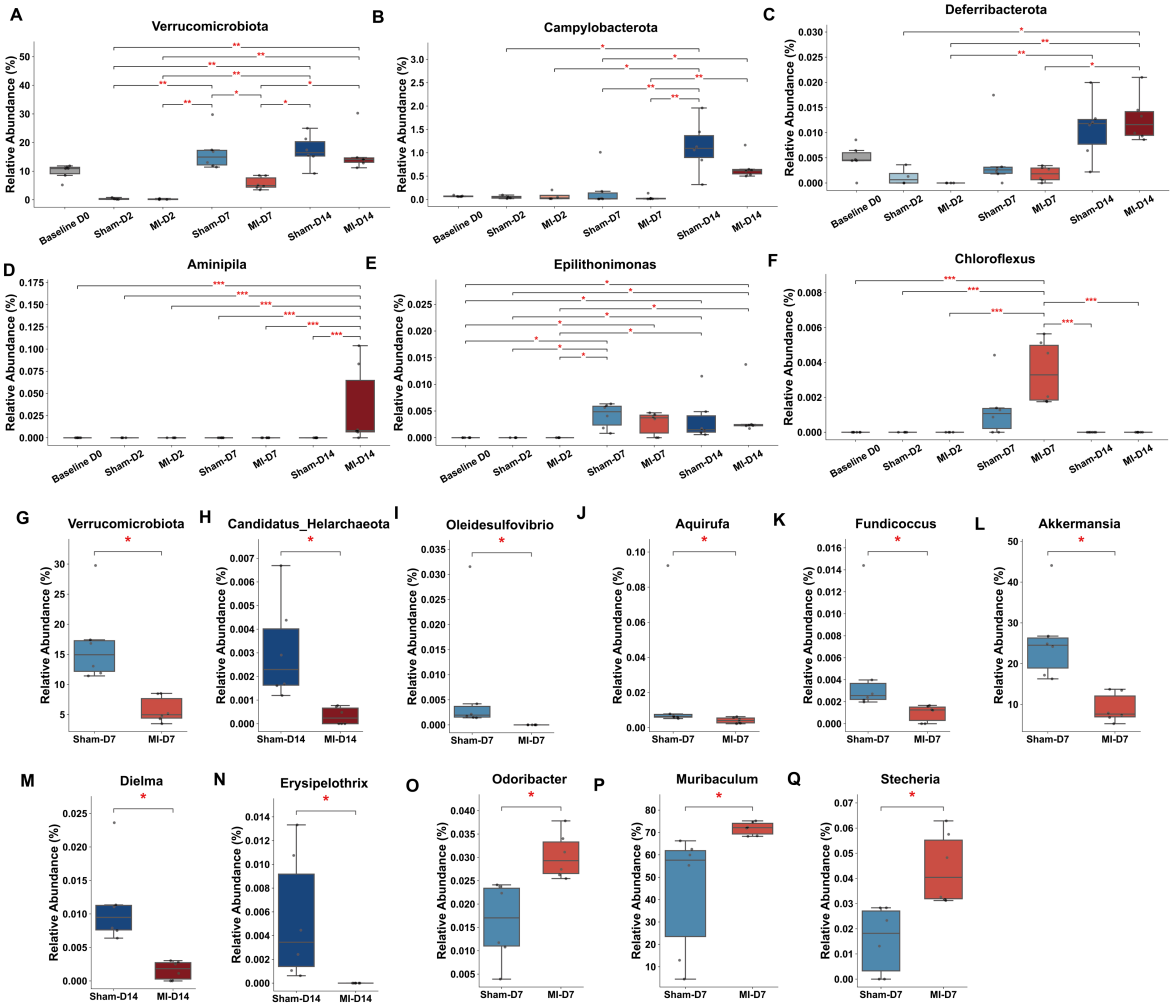
Taxonomic alterations in gut microbiota at the phylum and genus levels following myocardial infarction (MI) and sham surgery. **(A–C)** Relative abundance changes in key phyla across time points and treatment groups. **(A)** Verrucomicrobiota showed a sharp decline at Day 2 (D2), followed by gradual recovery by Day 14 (D14), maintaining higher levels in the sham group than in the MI group. **(B)** Campylobacterota and **(C)** Deferribacterota were significantly enriched at D14. **(D–F)** Genera exhibiting significant dynamic changes across groups and time. **(D)** Aminipila was highly abundant in the MI-D14 group. **(E)** Epilithonimonas showed increased abundance at D7 and D14. **(F)** Chloroflexus exhibited enrichment at D7 in both the MI and sham groups. **(G–H)** Significant phylum-level differences between MI and sham groups at specific time points. **(G)** Verrucomicrobiota was significantly lower in MI rats at D7. **(H)** Candidatus Helarchaeota showed reduced abundance in the MI group at D14. **(I–Q)** Differentially abundant genera between the MI and sham groups at D7 and D14. Genera including **(I)** Oleidesulfovibrio, **(J)** Aquirufa, **(K)** Fundicoccus, and **(L)** Akkermansia were significantly lower in the MI group at D7, while **(M)** Dielma and **(N)** Erysipelothrix experienced notable reductions by D14. In contrast, **(O)** Odoribacter, **(P)** Muribaculum, and **(Q)** Stecheria were significantly enriched in the MI group at D7. All statistical comparisons were based on Kruskal-Wallis tests followed by *post hoc* pairwise comparisons with FDR correction.

The phylum Verrucomicrobiota demonstrated the most pronounced dynamic shifts, undergoing near-complete depletion by Day 2 across both groups, followed by progressive recovery at Day 7 and subsequent enrichment by Day 14. Furthermore, its relative abundance remained consistently suppressed in the MI cohort compared to sham controls, reaching statistical significance at Day 7 (Figure 4A). Additional phyla that significantly expanded over the time course included Campylobacterota (Figure 4B), Deferribacterota (Figure 4C), and Bdellovibrionota, whereas fungal (Zoopagomycota) and archaeal (*Candidatus* Helarchaeota) populations displayed incomplete recovery trajectories, pointing to prolonged disruptions within the non-bacterial microbiome. Other phyla exhibiting significant temporal alterations included Thermodesulfobacteriota, Thermoproteota, Mucoromycota, Lentisphaerota, Spirochaetota, *Candidatus* Woesearchaeota, Aquificota, and Planctomycetota.

At the genus level, 179 of the 366 identified genera were significantly altered over time (FDR < 0.05), with the top 20 most dynamic genera detailed in Table 2. Notably, *Aminipila* exhibited a striking expansion, particularly within the MI group at Day 14 (Figure 4D). The genera *Epilithonimonas* (Figure 4E) and *Chloroflexus* (Figure 4F) expanded during both the subacute and recovery phases, while *Akkermansia* demonstrated a gradual rebound following its initial acute depletion. Conversely, *Cohnella* remained consistently suppressed across all observed time points.

**Table 2:** Significant changed genera across groups by metaproteins.

Phylum	Genus	Statistic	*P* value	FDR
Bacillota	Aminipila	33.07	1.02E-05	3.72E-03
Bacteroidota	Epilithonimonas	29.64	4.61E-05	4.22E-03
Chloroflexota	Chloroflexus	30.76	2.81E-05	4.22E-03
Verrucomicrobiota	Akkermansia	29.65	4.59E-05	4.22E-03
Bacillota	Cohnella	27.78	1.03E-04	4.77E-03
Bacillota	Fundicoccus	26.90	1.51E-04	4.77E-03
Bacillota	Caproicibacterium	26.78	1.59E-04	4.77E-03
Bacillota	Candidatus Merdiplasma	25.83	2.40E-04	4.77E-03
Bacteroidota	Candidatus Cryptobacteroides	25.86	2.37E-04	4.77E-03
Bacteroidota	Barnesiella	25.75	2.47E-04	4.77E-03
Bacteroidota	Alloprevotella	26.87	1.53E-04	4.77E-03
Bacteroidota	Aquirufa	28.37	8.00E-05	4.77E-03
Bacteroidota	Hymenobacter	25.78	2.45E-04	4.77E-03
Bacteroidota	Empedobacter	27.98	9.47E-05	4.77E-03
Pseudomonadota	Mesorhizobium	26.71	1.64E-04	4.77E-03
Pseudomonadota	Caballeronia	26.42	1.86E-04	4.77E-03
Pseudomonadota	Pseudoalteromonas	26.63	1.70E-04	4.77E-03
Pseudomonadota	Xanthomonas	27.17	1.34E-04	4.77E-03
Thermodesulfobacteriota	Oleidesulfovibrio	25.75	2.48E-04	4.77E-03
Thermodesulfobacteriota	Mailhella	25.56	2.69E-04	4.93E-03

Top 20 genera significant changed across groups by metaproteins.

Direct pairwise comparisons between the MI and sham groups pinpointed the specific taxa driving post-infarction microbial reprogramming. At the phylum level, Verrucomicrobiota was significantly diminished in the MI group at Day 7 (Figure 4G), and *Candidatus* Helarchaeota was significantly lower in the MI cohort at Day 14 (Figure 4H). At the genus level, several taxa including *Oleidesulfovibrio* (Figure 4I), *Aquirufa* (Figure 4J), *Fundicoccus* (Figure 4K), and *Akkermansia* (Figure 4L) were significantly depleted in MI rats at Day 7, while Dielma (Figure 4M) and Erysipelothrix (Figure 4N) experienced notable reductions by Day 14. In contrast, the genera *Odoribacter* (Figure 4O), *Muribaculum* (Figure 4P), and *Stecheria* (Figure 4Q) were significantly enriched in MI rats during the Day 7 subacute phase, implicating these taxa in active post-infarction remodeling.

Crucially, the absence of major phylum- or genus-level differences between the MI and sham groups at Day 2 strongly supports the hypothesis that these acute microbial shifts reflect a generalized physiological stress response to surgery, rather than an MI-specific pathology. Divergence between the cohorts primarily emerged at Day 7 and persisted into Day 14. The emergence of these distinct taxonomic biosignatures at these later time points, further implicating these specific organisms in the functional remodeling of the gut microbiota subsequent to myocardial injury.

### Functional remodeling of the gut microbiome after MI

**Figure 5:**
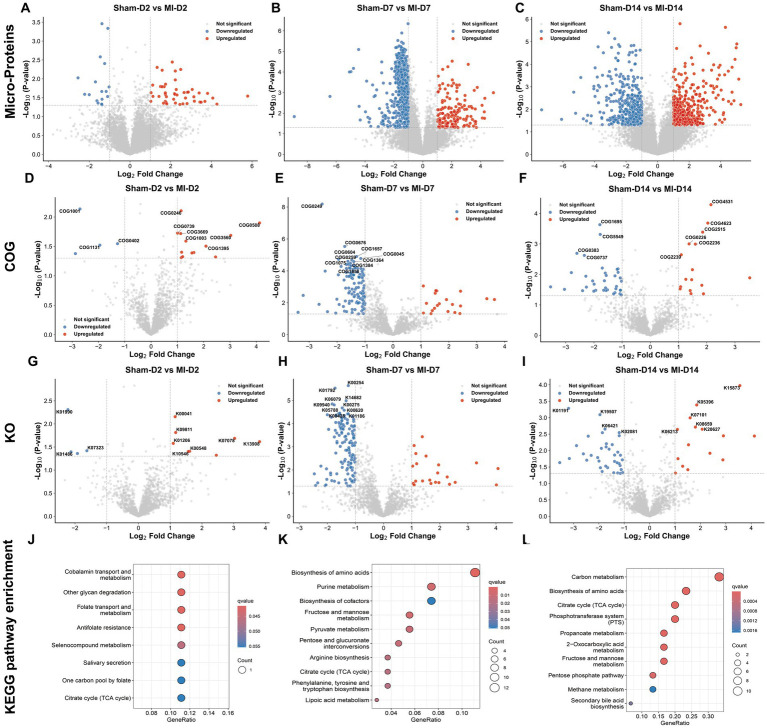
Protein-level and functional changes in the gut microbiome after myocardial infarction. **(A–C)** Volcano plots highlighting differentially abundant microbial proteins between the MI and sham groups at Day 2 **(A)**, Day 7 **(B)**, and Day 14 **(C)**. Red and blue dots indicate significantly upregulated and downregulated proteins, respectively (*p* < 0.05). **(D–F)** Volcano plots of differentially abundant Clusters of Orthologous Groups (COGs) at Day 2 **(D)**, Day 7 **(E)**, and Day 14 **(F)**. **(G–I)** Volcano plots of differentially abundant KEGG Orthology (KO) terms at Day 2 **(G)**, Day 7 **(H)**, and Day 14 **(I)**. **(J–L)** KEGG pathway enrichment bubble plots based on significantly altered KOs at Day 2 **(J)**, Day 7 **(K)**, and Day 14 **(L)**. The x-axis represents the GeneRatio, node size reflects the gene count, and color intensity indicates the *q*-value. These panels highlight the dynamic, time-resolved remodeling of gut microbial protein functions and metabolic pathways following myocardial infarction.

To characterize the functional remodeling of the gut microbiome specific to MI, we compared microbial protein expression profiles between the MI and sham groups at Days 2, 7, and 14. Differential expression analysis identified a comparatively modest 63 differentially abundant proteins at Day 2 (46 upregulated, 17 downregulated; Figure 5A). However, this divergence expanded to 1,249 proteins at Day 7 (149 upregulated, 1,100 downregulated; Figure 5B), before narrowing to 885 proteins at Day 14 (422 upregulated, 463 downregulated; Figure 5C). The sheer magnitude of these shifts confirms that while the acute phase is dominated by a shared surgical stress response, true MI-specific microbial functional perturbation peaks dramatically during the subacute phase.

Functional classification utilizing the COG database revealed marked, stage-dependent alterations in microbial protein functions. Specifically, 17, 137, and 52 COGs were significantly differentially abundant at Days 2, 7, and 14, respectively (*p* < 0.05; Figures 5D–F). At Day 2, the differentially expressed proteins were predominantly associated with nucleotide metabolism, cyclic di-GMP signaling, and carbohydrate utilization. Representative examples included COG1496 (EAL domain, c-di-GMP-specific phosphodiesterase class I, *p* = 0.0060), COG1001 (Adenine/N6-methyladenosine deaminase, *p* = 0.0073), and COG0246 (mannitol-1-phosphate/altronate dehydrogenase, *p* = 0.0078). These early alterations indicate limited initial divergence in nucleotide signaling and nutrient utilization between the ischemic and generalized surgical stress responses. By Day 7, the number of altered COGs expanded more than eight-fold relative to Day 2. Notable subacute changes included COG0249 (DNA mismatch repair ATPase MutS, *p* = 6.53 × 10^−9^), COG0676 (D-hexose-6-phosphate mutarotase, *p* = 2.95 × 10^−6^), and COG1657 (terpene cyclase SqhC, *p* = 1.26 × 10^−5^). Such widespread shifts point to an extensive, MI-driven remodeling of genome stability, central carbon metabolism, and secondary metabolite production. At Day 14, although the total number of differentially abundant COGs decreased substantially, significant functional shifts persisted. Persistent alterations included COG4531 (ABC-type Zn^2+^ transporter ZnuA, *p* = 5.14 × 10^−5^), COG4623 (lytic murein transglycosylase MltF, *p* = 2.07 × 10^−4^), and COG1695 (DNA-binding transcriptional regulator, PadR family, *p* = 2.33 × 10^−4^), highlighting ongoing adjustments in metal ion transport, transcriptional regulation, and cell wall remodeling as microbial function remains incompletely normalized during the recovery phase.

In parallel, KEGG Orthology (KO)-based analysis identified 12, 163, and 52 significantly altered KOs at Days 2, 7, and 14, respectively, further substantiating the time-dependent functional remodeling of microbial pathways (Figures 5G–I). At Day 2, notable modifications included K18891 (ATP-binding cassette, subfamily B, multidrug efflux pump; *p* = 0.0015), K24847 (23S rRNA 5-hydroxycytidine C2501 synthase; *p* = 0.0016), and K01990 (ABC-2 type transport system ATP-binding protein; *p* = 0.0049), reflecting an early, mild disturbances in membrane transport, RNA modification, and efflux systems. By Day 7, these functional alterations reached their zenith; K00254 (dihydroorotate dehydrogenase; *p* = 2.29 × 10^−6^), K13789 (geranylgeranyl diphosphate synthase type II; *p* = 2.86 × 10^−6^), and K01792 (glucose-6-phosphate 1-epimerase; *p* = 2.95 × 10^−6^) were among the most significantly affected, highlighting profound, disease-specific perturbations in pyrimidine biosynthesis, isoprenoid metabolism, and carbohydrate processing. By Day 14, differential KOs—such as K15873 (7beta-hydroxy-3-oxochol-24-oyl-CoA 4-desaturase; *p* = 0.00011), K11085 (ABC transporter MsbA; *p* = 0.00034), and K05396 (D-cysteine desulfhydrase; *p* = 0.0017)—suggested a transition toward partial stabilization, alongside a persistent adjustments in secondary bile acid metabolism, membrane transport, and amino acid degradation.

Subsequent KEGG pathway enrichment analyses of the differentially abundant KOs further underscored the stage-specific nature of this microbial metabolic remodeling (Figures 5J–L). At Day 2, the enrichment of Cobalamin transport and metabolism, Folate transport and metabolism, and other glycan degradation pathways indicated early disturbances in vitamin transport, cofactor metabolism, and glycan degradation. By Day 7, pathways encompassing Biosynthesis of amino acids, Purine metabolism, and Fructose and mannose metabolism were significantly enriched, reflecting an extensive reprogramming of carbohydrate utilization, nucleotide biosynthesis, and amino acid metabolism unique to the post-infarction environment. At Day 14, the enrichment of Carbon metabolism, Citrate cycle (TCA cycle), and Phosphotransferase system (PTS) suggested a partial recovery of metabolic activity and the progressive stabilization of energy homeostasis.

Collectively, these findings demonstrate that MI triggers a highly dynamic and phase-specific reprogramming of both microbial protein expression and broader metabolic functions. The acute phase is characterized by relatively minor MI-specific deviations from the shared surgical stress response, whereas the subacute phase represents the undeniable peak of targeted metabolic reorganization and stress adaptation. Finally, the recovery phase demonstrates a partial restoration of energy metabolism amidst persistent functional disturbances. These results robustly underscore the remarkable plasticity of the gut microbiome and suggest that specific microbial functional signatures —particularly those emerging during the subacute window—may actively shape host recovery trajectories, offering potential targets for microbiota-based therapeutic strategies.

### Temporal patterns of host proteins

In addition to microbial proteins, 729 host-derived proteins were robustly quantified in fecal samples, providing critical readouts of epithelial turnover, immune activity, and barrier function. Differential expression analysis revealed distinct, time-dependent patterns that closely paralleled the microbial functional shifts.

**Figure 6:**
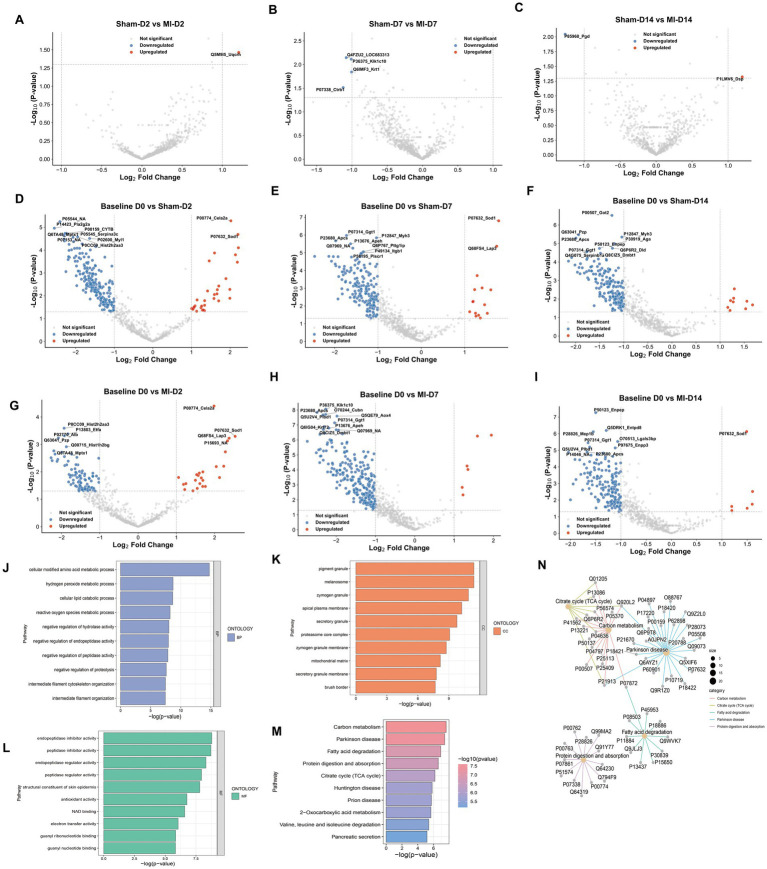
Dynamics of host-derived proteins and functional enrichment following myocardial infarction (MI). **(A–C)** Volcano plots showing differentially expressed host proteins between the MI and sham groups at Day 2 (D2), Day 7 (D7), and Day 14 (D14). Red dots represent significantly upregulated proteins in the MI group, and blue dots represent downregulated proteins (*p* < 0.05). **(D–I)** Volcano plots of host protein changes relative to baseline (D0) at each specific time point: Sham-D2 **(D)**, Sham-D7 **(E)**, Sham-D14 **(F)**, MI-D2 **(G)**, MI-D7 **(H)**, and MI-D14 **(I)**. **(J–L)** Bar plots detailing Gene Ontology (GO) enrichment of the differentially expressed host proteins in the MI-D7 group. **(J)** Top enriched Biological Process (BP) terms; **(K)** Cellular Component (CC) terms; **(L)** Molecular Function (MF) terms. **(M)** KEGG pathway enrichment bar plot for significantly altered host proteins in MI-D7. **(N)** Cnetplot visualizing the complex network of enriched KEGG pathways and their associated host proteins in MI-D7.

At Day 2, only two host proteins (1 upregulated, 1 downregulated) were significantly altered in the MI group compared to sham controls, including the upregulated ubiquinol-cytochrome c reductase hinge protein (Uqcrh) (Figure 6A). These subtle alterations likely represent a highly localized, non-specific epithelial stress response. However, by Day 7, the inter-group difference expanded, with 11 proteins emerging as significantly altered (1 upregulated, 10 downregulated). Notably, several of these proteins—including LOC683313, Klk10, Krt1, and Ctrb1, which are heavily implicated in epithelial defense, digestive function, and tissue integrity—were strongly downregulated (Figure 6B). This targeted proteomic suppression confirms that the subacute phase represents the specific peak of the host intestinal response directly attributable to myocardial injury. By Day 14, the inter-group divergence narrowed to five altered proteins (2 upregulated, 3 downregulated), including the downregulated Pgd and the upregulated Dsp (Figure 6C), indicating a partial normalization of host proteomic profiles alongside persistent metabolic and structural adjustments (Figure 6C).

To further disentangle the surgical stress response from MI-induced pathology and capture the broader scale of these shifts, we longitudinally compared fecal protein profiles at each postoperative time point against baseline (Day 0). In the sham group, the magnitude of differential expression peaked acutely at Day 2 (241 proteins; Figure 6D), followed by a gradual decline at Day 7 (213 proteins; Figure 6E) and Day 14 (197 proteins; Figure 6F). The vast majority of these postoperative changes represented a downregulation (e.g., 211 out of 241 proteins at Day 2), consistent with a generalized physiological suppression and redirection of intestinal resources following surgical trauma.

In stark contrast, while the MI group also exhibited an acute upregulation at Day 2 (115 proteins; Figure 6G), its differential expression profile intensified substantially over time. The MI group reached its absolute peak of broad proteomic alteration at Day 7 (288 proteins compared to baseline; Figure 6H). Crucially, the overwhelming majority of these subacute MI-associated proteins (281 out of 288) were heavily downregulated, pointing to a profound, disease-specific suppression of intestinal and metabolic functions. By Day 14, 198 proteins remained altered (Figure 6I), maintaining a predominantly downregulated pattern (189 downregulated) that signifies an ongoing, yet incomplete, recovery process.

Functional enrichment analysis of the MI cohort at Day 7 confirmed this subacute window as the peak of metabolic stress and adaptive intestinal remodeling. Enriched GO Biological Process terms included *cellular modified amino acid metabolic process*, *hydrogen peroxide metabolic process*, and *cellular lipid catabolic process* (Figure 6J). Cellular Component terms were notably enriched for granule-associated proteins such as *pigment granule*, *melanosome*, and *zymogen granule* (Figure 6K), while Molecular Function terms were dominated by *endopeptidase inhibitor activity* and *peptidase inhibitor activity*, indicating the active modulation of epithelial proteolysis and defense (Figure 6L). KEGG pathway analysis further highlighted the significant involvement of *Carbon metabolism*, *Fatty acid degradation*, *Protein digestion and absorption*, and *the Citrate cycle (TCA cycle)* (Figure 6M). These complex regulatory interactions were visualized utilizing a cnetplot (Figure 6N).

Collectively, these findings demonstrate that while surgery induces an immediate, generalized host physiological suppression, myocardial infarction triggers a delayed, far more profound intestinal metabolic reprogramming and targeted protein downregulation peaking at Day 7, followed by signs of partial, yet incomplete, functional recovery by Day 14.

### Integrated microbe–host coexpression networks

To systematically explore the coordinated functional interactions between gut microbial and host proteins following MI, we applied weighted gene co-expression network analysis (WGCNA) to the integrated metaproteomic dataset. A soft-thresholding power of 22 was established to achieve an approximate scale-free network topology (Supplementary Figure S5A). Subsequent hierarchical clustering of the topological overlap matrix generated several distinct coexpression modules, visualized as distinct branches within the resulting dendrogram (Supplementary Figure S5B). Each module represents a highly interconnected cluster of proteins exhibiting tightly correlated abundance profiles across the biological samples.

**Figure 7:**
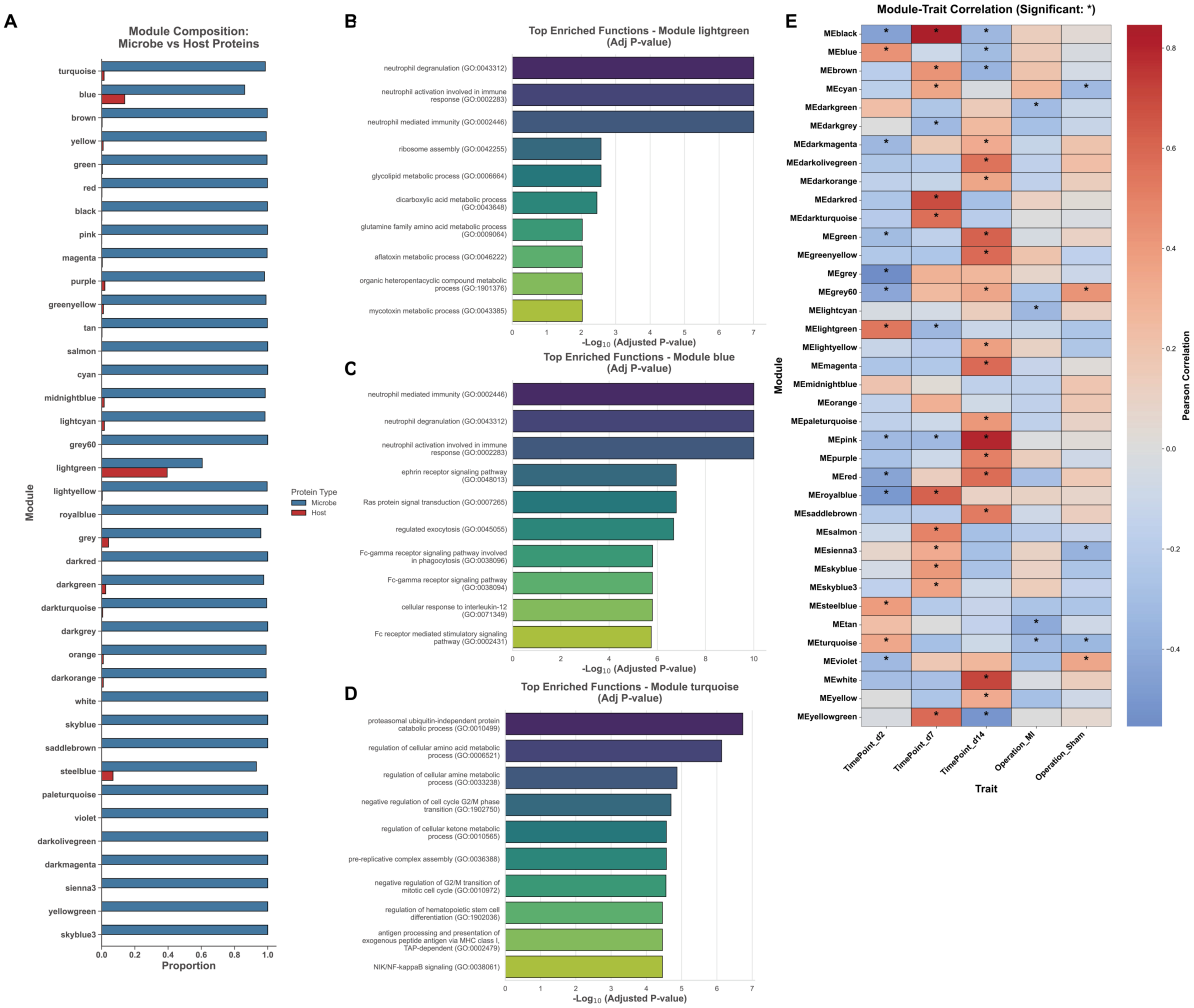
Microbe–host coexpression network analysis based on integrated metaproteomics data. **(A)** Bar plot showing the quantitative proportion of host- and microbe-derived proteins within each module, revealing their functional composition and taxonomic origin. **(B–D)** Top enriched functions for the selected representative modules: lightgreen **(B)**, blue **(C)**, and turquoise **(D)**. **(E)** Heatmap representing module–trait correlations. Rows denote the identified modules, and columns correspond to clinical traits and time points. Significant associations highlight coordinated shifts in module expression in relation to disease status and temporal progression.

Detailed analysis of module composition revealed a clear functional dichotomy: certain clusters were predominantly driven by host proteins, whereas others were heavily enriched in microbial proteins (Figure 7A). For instance, the *lightgreen* module contained the highest proportion of host-derived proteins, suggesting its central involvement in host-driven biological processes. Specifically, functional enrichment analysis revealed that this module is primarily associated with *neutrophil degranulation*, *neutrophil activation involved in immune response*, *neutrophil mediated immunity* (Figure 7B). In contrast, modules such as *blue* and *turquoise* were predominantly microbial in origin, although the blue module retained a minor fraction of host proteins (Figure 7A). The turquoise module, for example, was strongly enriched for *proteasomal ubiquitin-independent protein catabolic process*, *regulation of cellular amino acid metabolic process*, and *regulation of cellular amine metabolic process* (Figure 7D), reflecting critical microbial metabolic activity operating throughout the infarction and recovery phases. Interestingly, the blue module was heavily enriched for *immune and signaling functions*, *including neutrophil mediated immunity* and the *ephrin receptor signaling pathway* (Figure 7C).

Furthermore, module–trait correlation analysis illuminated biologically significant associations with both the experimental condition (MI versus sham) and the temporal progression (Days 2, 7, and 14) (Figure 7E). Within the MI cohort (Operation_MI), specific modules—including *darkgreen*, and *turquoise*—were significantly negatively correlated with disease status (Figure 7E). Rather than merely representing a generic downregulation, functional annotation of these specific clusters indicates a profound, disease-specific suppression of core biological functions, such as the amino acid and amine metabolic processes defining the turquoise module (Figure 7D). Conversely, modules such as *violet* and *grey60* demonstrated significant positive associations exclusively with the sham controls (Operation_Sham) (Figure 7E), underscoring group-specific baseline patterns that operate independently of myocardial infarction.

Finally, temporal correlations unveiled highly dynamic, stage-dependent host–microbiome interactions that tightly align with our differential expression findings. For example, the host-dominated *lightgreen* module—enriched for acute immune and neutrophil responses (Figure 7B)—was significantly positively correlated with the Day 2 time point but exhibited a strong, significant negative correlation at Day 7 (Figure 7E). This exact trajectory confirms a transient, acute activation of host responses tied to initial surgical stress, which then rapidly diminishes and shifts into profound metabolic suppression as the MI pathology progresses into the subacute phase. Collectively, these complex coexpression patterns underscore the profound plasticity of the gut ecosystem and highlight the tightly coordinated synchronization of host and microbial processes traversing the acute, subacute, and recovery phases of MI.

## Discussion

In the present study, we utilized time-resolved fecal metaproteomics to characterize the dynamic interactions between the gut microbiota and host responses following MI in a rat model. By integrating microbial and host proteomic profiles across the acute, subacute, and recovery phases, we demonstrated that following an initial physiological response to surgical trauma, MI induces profound, stage-specific alterations in microbial composition, functional activity, and host intestinal physiology. These findings yield novel mechanistic insights into the gut–heart axis and highlight critical therapeutic windows for microbiota-targeted interventions aimed at supporting post-MI recovery.

During the immediate postoperative phase (Day 2), microbial diversity declined precipitously across both the MI and sham cohorts. This acute collapse is characteristic of a generalized surgical stress response. Previous studies have robustly demonstrated that perioperative interventions—including general anesthesia and transient fasting—rapidly reduce microbial diversity (Joshi et al., 2026). Concurrently, laparotomy and surgical manipulation acutely disrupt intestinal motility (often inducing transient postoperative ileus) (Mazzotta et al., 2020) and induce mucosal ischemia and oxygenation shifts that decimate obligate anaerobes (Zheng et al., 2023; Oh, 2025). Together, these factors explain the shared acute dysbiosis. Nevertheless, the concurrent elevation of host proteins associated with inflammation and oxidative stress, notably calprotectin and myeloperoxidase, underscores how this acute surgical stress rapidly disrupts gut microbial homeostasis and intestinal barrier integrity, potentially increasing intestinal permeability and creating a vulnerable systemic environment (Nishiwaki et al., 2022; O’Riordan et al., 2022; Proffitt et al., 2022; Yan et al., 2022). However, the critical pathophysiological divergence emerges at Day 7. While the sham cohort rapidly restores microbial homeostasis following the resolution of surgical trauma, the MI cohort exhibits sustained functional dysbiosis. We postulate that this prolonged MI-specific disruption is driven by the onset of hemodynamic compromise—specifically, reduced cardiac output resulting in chronic intestinal hypoperfusion—coupled with sustained sympathetic hyperactivation and systemic cytokine release from the infarcted myocardium (Lorenz and Fink, 2002; Hills et al., 2019; Han et al., 2021; Bijla et al., 2024). These systemic sequelae create a persistently hostile microenvironment in the gut, precluding the restoration of baseline commensal populations.

The subacute phase (Day 7) represented the critical turning point where MI-specific pathology clearly diverged from the shared surgical stress recovery, characterized by extensive metabolic reprogramming in both the microbiome and the host. Microbial proteomes in the MI cohort became uniquely enriched for pathways driving carbohydrate metabolism, nucleotide biosynthesis, and oxidative stress adaptation. In stark contrast, host proteomes exhibited a profound disease-specific suppression, characterized by the heavy downregulation of proteins implicated in epithelial defense, tissue integrity, and digestive function. WGCNA confirmed the presence of highly synchronized microbial–host coexpression modules, suggesting a coordinated adaptation to systemic metabolic stress. Taxonomic shifts were equally pronounced: *Odoribacter* and *Muribaculum* expanded significantly, while *Erysipelothrix* and *Dielma* declined. Notably, *Odoribacter*—a butyrate-producing anaerobe—has been implicated in both epithelial repair and pro-inflammatory signaling, while *Muribaculum*, a core rodent gut taxon involved in polysaccharide degradation and mucosal immunity, may exert similarly dual, context-dependent effects. These findings underscore the inherent complexity of microbial responses post-MI, where protective and pathogenic processes actively coexist. Functional pathway enrichment further revealed significant shifts toward the TCA cycle and amino sugar metabolism, indicating a partial restoration of microbial energy metabolism running in parallel with host recovery (Guo et al., 2024). This functional convergence strongly supports the concept of a microbiota–metabolite–myocardium regulatory axis, whereby microbial metabolic reprogramming intimately mirrors and potentially modulates host physiology during the recovery phase.

By the recovery phase (Day 14), both the microbial and host proteomes demonstrated partial normalization. *Akkermansia* abundance and short-chain fatty acid (SCFA) biosynthetic proteins began to rebound, while essential host barrier proteins, such as mucin-2 and ZO-1, increased in abundance. A recovery-associated protein module enriched for antioxidant defenses and SCFA metabolism further emphasized the potential role of microbial metabolites and host protective pathways in physiological restoration. Nevertheless, residual proteomic alterations indicated that gut and host responses remained incompletely normalized, highlighting the profound, long-lasting impact of MI on intestinal homeostasis.

Our results significantly extend previous cardiovascular microbiome studies, which have primarily relied on 16S rRNA sequencing or metagenomics to describe compositional shifts. Unlike these DNA-based methods, metaproteomics directly quantifies functional proteins, thereby capturing real-time microbial activity and host responses. Importantly, we identified distinct, temporally defined phases of shared surgical dysbiosis, MI-specific metabolic reprogramming, and subsequent recovery; this suggests that microbiota-targeted therapeutic strategies should be precisely tailored to specific windows of MI progression, particularly during the subacute phase when maladaptive host–microbe interactions appear to peak (Gagné et al., 2022). WGCNA further revealed distinct host- and microbe-enriched protein modules tightly associated with specific MI stages and recovery trajectories. These coexpression clusters vividly highlight synchronized host–microbiota responses and likely represent key regulatory networks mediating immune modulation, metabolic adaptation, and tissue remodeling following infarction.

Several limitations of this study should be acknowledged. First, as this research was conducted in a controlled rat model, direct translation to human pathophysiology requires rigorous validation in clinical cohorts, where variables such as diet, comorbidities, and polypharmacy inevitably influence microbiome dynamics. Second, fecal metaproteomics primarily captures luminal proteins and may not fully encompass mucosal-adherent or broader systemic changes. Furthermore, interpreting host proteins in fecal samples warrants caution due to severe proteolytic degradation within the digestive tract; therefore, the identified host proteins may reflect a combination of active epithelial secretion and residual cellular debris. Third, while this study generates robust functional hypotheses, the findings largely remain computational predictions. Independent experimental validation—such as targeted metabolomics (e.g., GC–MS) for absolute SCFA quantification and Western blotting or ELISA for key host tissue proteins—will be required to definitively substantiate these functional pathways. Finally, while our WGCNA successfully uncovered strong host–microbe correlations, these observational data merely suggest potential regulatory networks; definitive causality remains to be established.

Future work should prioritize mechanistic studies utilizing germ-free or antibiotic-depleted MI models to definitively test causality within these host–microbe interactions. Targeted interventions with *Akkermansia*-derived SCFAs or microbial glyoxylate-cycle intermediates could help clarify their respective protective versus maladaptive effects on recovery. In parallel, longitudinal human studies integrating metaproteomics with comprehensive metabolomics and immune profiling will be critical to distinguishing universal, core microbial signatures from individualized responses relevant to MI prognosis and precision therapy.

In conclusion, time-resolved metaproteomics effectively demonstrates that following acute surgical trauma, MI drives highly dynamic and coordinated alterations in both the gut microbiota and the host proteome. These alterations progress sequentially from shared acute dysbiosis to disease-specific subacute metabolic reprogramming and, ultimately, partial functional recovery. These findings firmly establish the gut–heart axis as a critical determinant of MI outcomes and suggest that appropriately timed microbiota-targeted interventions—particularly those deployed during the vulnerable subacute phase—hold significant promise for improving cardiovascular recovery.

## Data Availability

The raw data generated in this study can be found in the National Genomics Data Center (https://ngdc.cncb.ac.cn/), accession number PRJCA042260.

## Supplementary material

Supplementary Figure S1 Longitudinal echocardiographic assessment of cardiac function. Echocardiographic evaluation of Left Ventricular Ejection Fraction (LVEF) and Fractional Shortening (LVFS) at Days 2, 7, and 14 post-surgery. The data demonstrate a significant and sustained reduction in both LVEF **(A)** and LVFS **(B)** in the myocardial infarction (MI) group compared to the sham-operated controls, confirming successful induction of cardiac dysfunction.

Supplementary Figure S2 Histological evaluation of myocardial injury via H&E staining. Representative images of Hematoxylin and Eosin (H&E)-stained heart cross-sections at the study endpoint (Day 14). The MI group exhibits marked structural tissue remodeling, extensive myocyte necrosis, and dense inflammatory cell infiltration within the infarcted myocardium, whereas the sham group displays normal cardiac architecture.

Supplementary Figure S3 Histological and quantitative assessment of myocardial fibrosis and infarct size. **(A)** Representative Masson’s trichrome-stained whole-heart cross-sections from the sham and MI groups at Day 14 post-surgery. Extensive transmural collagen deposition (stained blue) and ventricular wall thinning are clearly evident in the MI cohort, contrasting with the normal architecture of the sham controls. Scale bars = 1 mm. **(B)** Quantification of the collagen volume fraction (%), demonstrating a significant increase in fibrosis in the MI group relative to the sham group (**p* < 0.05). **(C)** Quantitative measurement of the infarct size (%) specifically within the MI cohort. Bar graphs represent the mean ± standard deviation (SD), with individual data points shown.

Supplementary Figure S4 Global functional annotation of the fecal metaproteome. **(A)** Overview of the relative abundance and distribution of primary KEGG functional categories across the identified microbial proteins. **(B)** Relative distribution of these primary KEGG functional categories across individual samples, demonstrating a consistent representation of core metabolic activities. **(C)** Total number of microbial proteins successfully mapped to distinct Clusters of Orthologous Groups (COG) functional categories.

Supplementary Figure S5 Construction of the weighted gene co-expression network (WGCNA). **(A)** Analysis of network topology for various soft-thresholding powers. A soft-thresholding power of 22 was selected to achieve an approximate scale-free network topology. **(B)** Hierarchical clustering dendrogram of the topological overlap matrix (TOM), illustrating the generation of distinct protein coexpression modules (visualized as distinct colored branches) across the integrated metaproteomic dataset.
