# Supplementary figures and images for: Dynamic remodeling of the gut microbiome and host responses after myocardial infarction revealed by longitudinal metaproteomics
Source: Front Microbiol. 2026 Jul 10;17:1826248. doi: 10.3389/fmicb.2026.1826248 (PMC13396157; doi:10.3389/fmicb.2026.1826248)

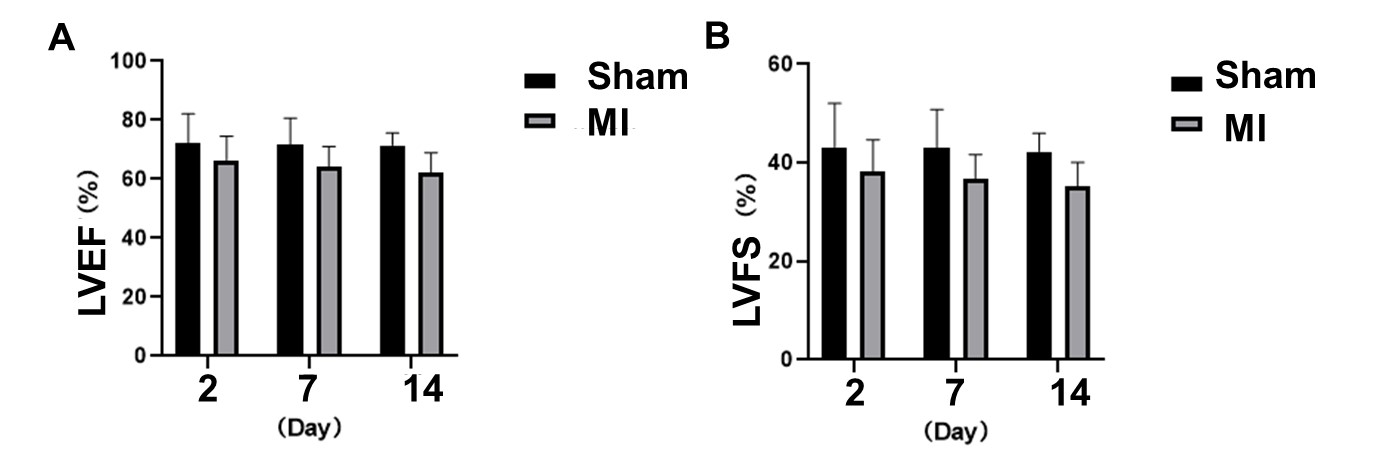

Supplement: Supplementary Figure S1 — Longitudinal echocardiographic assessment of cardiac function. Echocardiographic evaluation of Left Ventricular Ejection Fraction (LVEF) and Fractional Shortening (LVFS) at Days 2, 7, and 14 post-surgery. The data demonstrate a significant and sustained reduction in both LVEF (A) and LVFS (B) in the myocardial infarction (MI) group compared to the sham-operated controls, confirming successful induction of cardiac dysfunction. [file Image_1.jpeg]

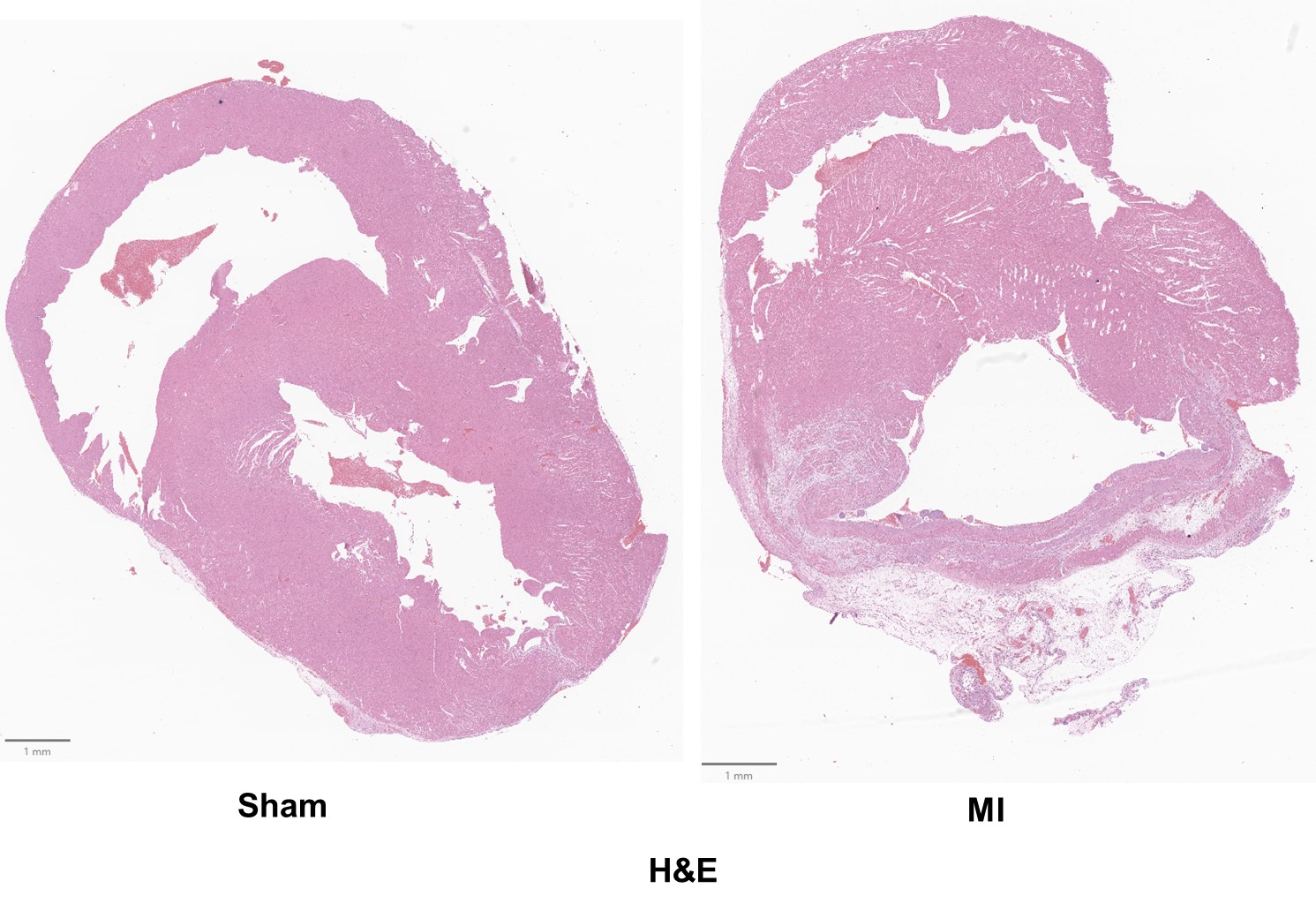

Supplement: Supplementary Figure S2 — Histological evaluation of myocardial injury via H&E staining. Representative images of Hematoxylin and Eosin (H&E)-stained heart cross-sections at the study endpoint (Day 14). The MI group exhibits marked structural tissue remodeling, extensive myocyte necrosis, and dense inflammatory cell infiltration within the infarcted myocardium, whereas the sham group displays normal cardiac architecture. [file Image_2.jpeg]

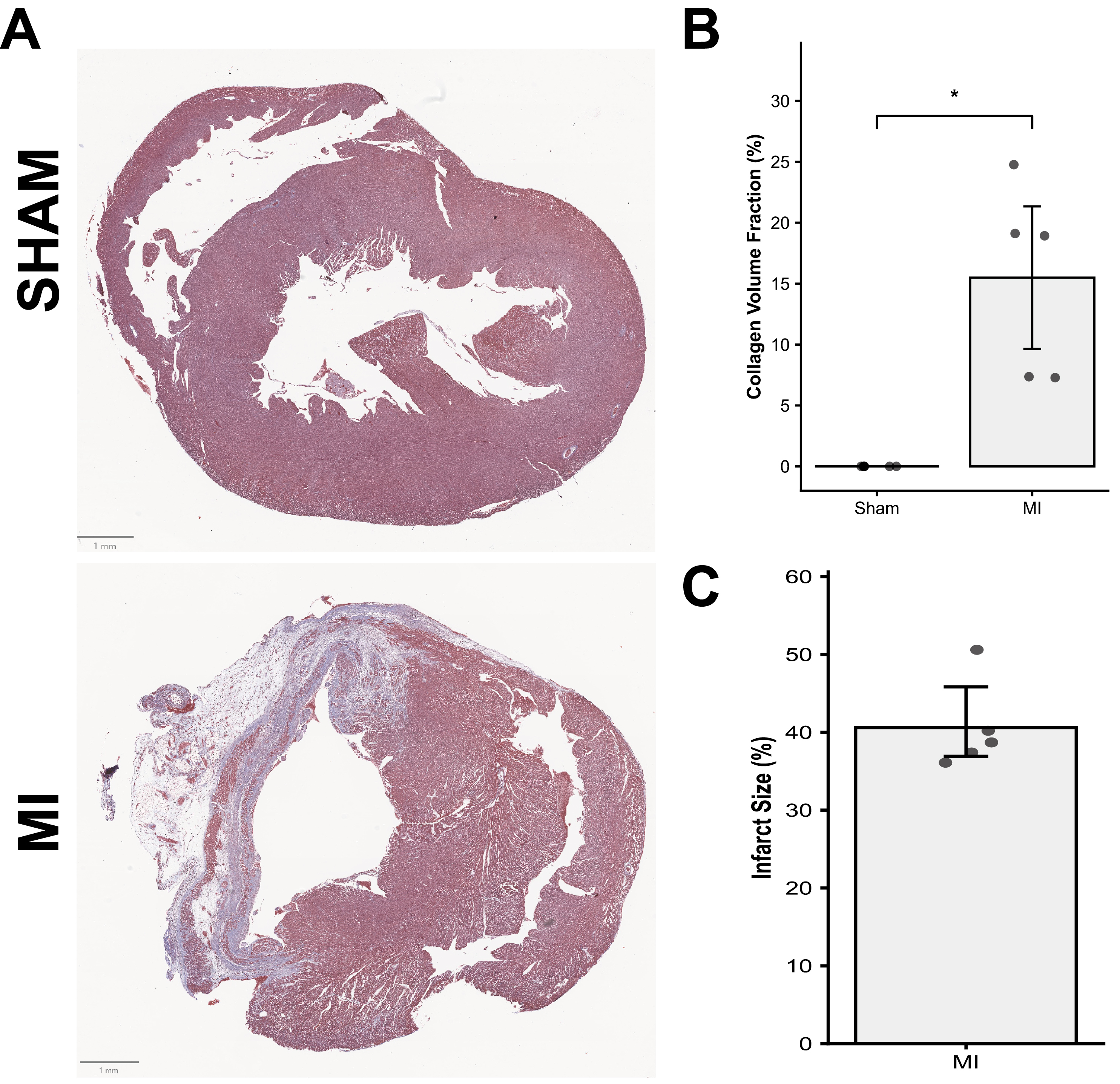

Supplement: Supplementary Figure S3 — Histological and quantitative assessment of myocardial fibrosis and infarct size. (A) Representative Masson’s trichrome-stained whole-heart cross-sections from the sham and MI groups at Day 14 post-surgery. Extensive transmural collagen deposition (stained blue) and ventricular wall thinning are clearly evident in the MI cohort, contrasting with the normal architecture of the sham controls. Scale bars = 1 mm. (B) Quantification of the collagen volume fraction (%), demonstrating a significant increase in fibrosis in the MI group relative to the sham group (*p < 0.05). (C) Quantitative measurement of the infarct size (%) specifically within the MI cohort. Bar graphs represent the mean ± standard deviation (SD), with individual data points shown. [file Image_3.jpeg]

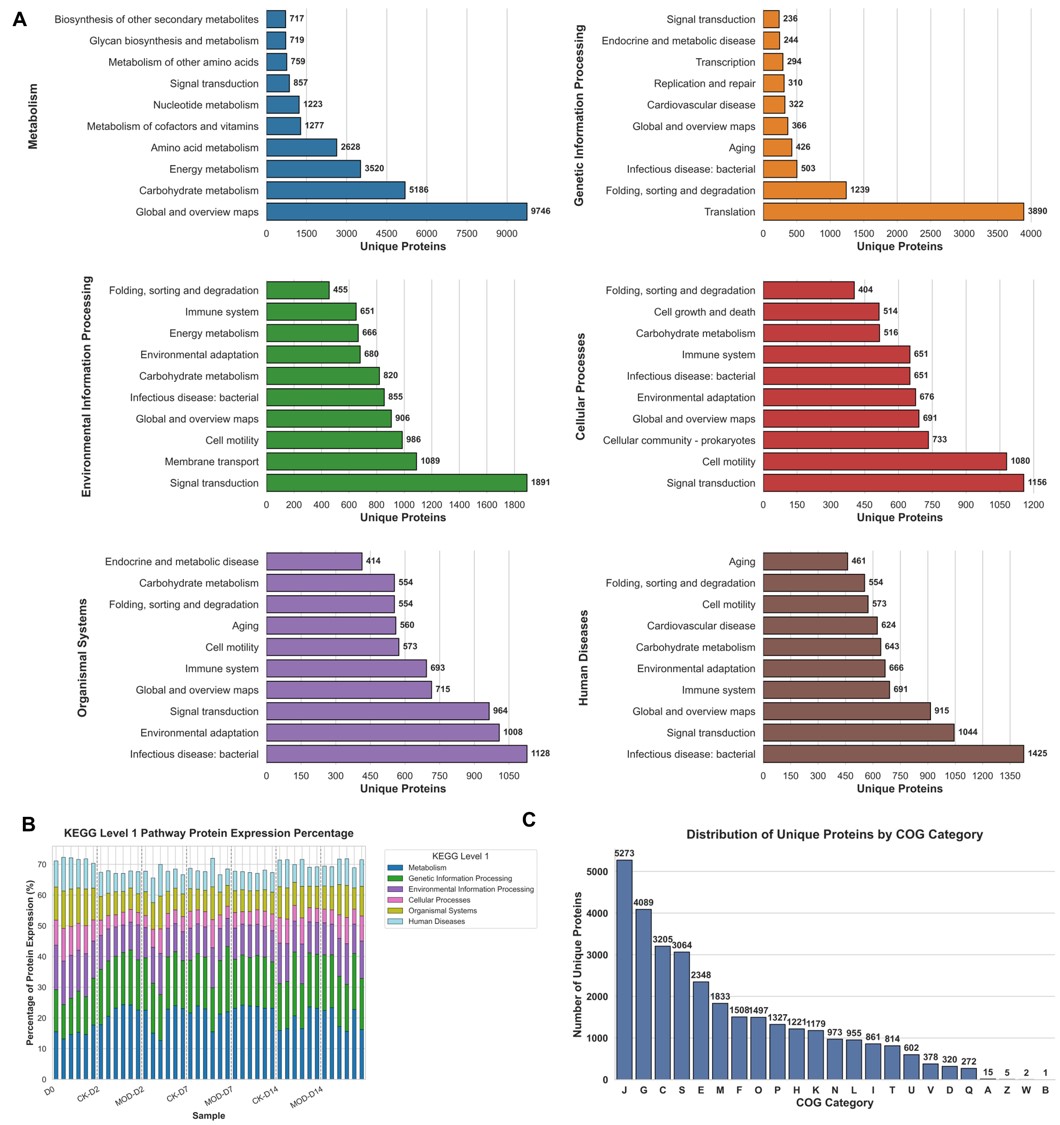

Supplement: Supplementary Figure S4 — Global functional annotation of the fecal metaproteome. (A) Overview of the relative abundance and distribution of primary KEGG functional categories across the identified microbial proteins. (B) Relative distribution of these primary KEGG functional categories across individual samples, demonstrating a consistent representation of core metabolic activities. (C) Total number of microbial proteins successfully mapped to distinct Clusters of Orthologous Groups (COG) functional categories. [file Image_4.jpeg]

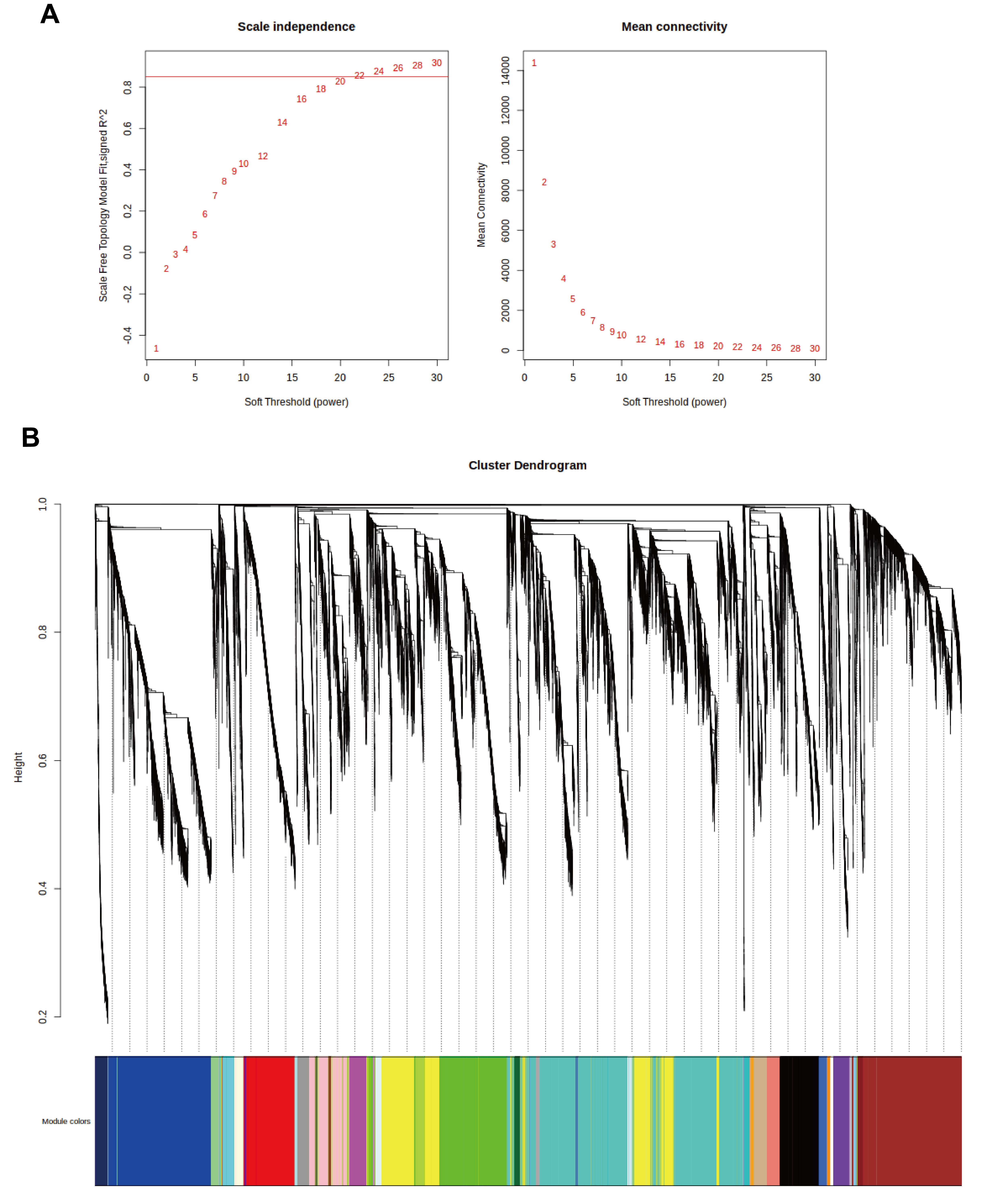

Supplement: Supplementary Figure S5 — Construction of the weighted gene co-expression network (WGCNA). (A) Analysis of network topology for various soft-thresholding powers. A soft-thresholding power of 22 was selected to achieve an approximate scale-free network topology. (B) Hierarchical clustering dendrogram of the topological overlap matrix (TOM), illustrating the generation of distinct protein coexpression modules (visualized as distinct colored branches) across the integrated metaproteomic dataset. [file Image_5.jpeg]
